# Foreign body aspiration in a tertiary Syrian centre: A 7-year retrospective study

**DOI:** 10.1016/j.heliyon.2021.e06537

**Published:** 2021-03-17

**Authors:** Fatema Mohsen, Batoul Bakkar, Sara Melhem, Roula Altom, Bisher Sawaf, Imad Alkhija, Louei Darjazini Nahas

**Affiliations:** a Faculty of Medicine, Syrian Private University, Damascus, Syria; b Department of Internal Medicine, Faculty of Medicine, Syrian Private University, Damascus, Syria; c Faculty of Medicine, American University of Beirut Medical Centre Beirut, Lebanon; d Internal Medicine Department, Hamad General Hospital, Hamad Medical Corporation, Doha, Qatar; e Department of Otorhinolaryngology, Faculty of Medicine, Al Mouwasat University Hospital, Syria; f Department of Surgery Division of Otorhinolaryngology, Faculty of Medicine, Syrian Private University, Damascus, Syria

**Keywords:** Paediatric, Foreign body aspiration, Bronchoscopy, Syria, War, Children

## Abstract

**Background:**

Paediatric foreign body aspiration constitutes significant lethal sequela worldwide. This is the first descriptive Syrian study that aims to describe the epidemiology of foreign body aspiration in a tertiary centre in Syria.

**Methods:**

This is a retrospective cohort study conducted at the Children's University Hospital, Damascus, from 2011 to 2018 during the Syrian crisis. The children underwent rigid bronchoscopy at Al Mouwasat University hospital, the only properly equipped hospital to perform paediatric rigid bronchoscopy in Damascus. We included all cases with complete medical records of children under the age of 13 years with positive bronchoscopy findings of foreign body aspiration. The records of patients were examined, and data extracted included physical examination, CXR reports, bronchoscopy reports, and complications. Statistical package for social sciences 25.0 program for Windows was used to report frequencies, percentages, means, medians, and standard deviations.

**Results:**

Of 560 children diagnosed with foreign body aspiration, the peak incidence was at the age of 1–3 years 376 (67.2%). Most patients presented with an explicit history of inhalation 453 (80.9%). The most frequent clinical findings were dyspnoea 320 (57.1%), wheezing 308 (55%), and chest retraction 209 (37.35%). Hyperinflation 260 (46.4%) followed by pulmonary infiltration 197 (35.2%) were the most common abnormal radiological findings. Seeds 273 (48.8%) were the most frequent foreign body extracted by rigid bronchoscopy. The right main bronchus 255 (40.2%) was the most frequent site of foreign body lodgement. Lobar pneumonia 16 (2.8%) was the commonest complication of foreign body aspiration.

**Conclusion:**

Foreign body aspiration is a major public health problem in Syria. The child's welfare must be our paramount concern. To prevent this accident, we should address a change in raising public health awareness with regards to appropriate food and eating habits. This would limit hazardous complications.

## 1 Introduction

Paediatric foreign body aspiration (FBA) is a serious condition leading to lethal sequela [1]. Consequences can be fatal, with patients suffering from anoxic brain injury (2.2%) and mortality (1.8%) [2]. Paediatric FBA is defined as asphyxia, suffocation, or inhalation of a solid substance into the airway tract at the level of the glottis, larynx, trachea, or bronchi by a child. FBA manifests as cough, wheeze, and decreased breath sounds (classical triad), and delayed diagnosis can lead to respiratory complications. The main risk factors for FBA include children (x < 2 years), and organic foreign body [3]. In such cases, clinical features, physical examination, chest x-ray (CXR) are guidelines for otorhinolaryngologists; however, bronchoscopy for treatment and diagnosis is essential in FBA. Flexible bronchoscopy remains the gold standard for FBA. Indications for bronchoscopy include, airway compression, recurrent and complicated pneumonia, FBA, interstitial lung disease, endoscopic intubation, caustic ingestion, and bronchiectasis, with stridor and wheeze as the most common indications [4]. More recent reports have suggested using computed tomography (CT) to prevent unnecessary bronchoscopy, decrease operational risks and costs without increasing misdiagnosis [5, 6]. This has not been implemented into our protocols to date. Although the diagnostic yield of CT scan is superior to that of CXR (sensitivity = 100% and specificity = 66.7%), it is the last resolve as it contradicts the contemporary approach for minimizing ionizing irradiation exposure, particularly with regards to children [7].

It is important to mention that the majority of studies on the incidences of FBA were carried out in countries with high to intermediate economic status [8, 9, 10]. Recent studies discussed the use of CT scans over CXR to avoid unnecessary bronchoscopy procedures and the benefits of using flexible bronchoscopy over rigid bronchoscopy [10, 11, 12]. No studies have given an insight into the management of FBA in war-ridden regions where resources are reduced to a bare minimum. Syria shows scarcity of data around FBA and bronchoscopy; however, literature search revealed a case report on laryngeal hirudiniasis [13]. The condition of leech (*Hirudinea* a subclass of predatory worms) attachment to the larynx is known as Laryngeal hirudiniasis.

The Syrian crisis has affected the health care system catastrophically: hospitals destroyed, doctors and nurses immigrating, children not vaccinated, and a huge demand for medical supplies. Millions of Syrian children have been displaced since the crisis began, growing up knowing nothing but war, terror, and displacement [14].

This is the first study that aims to provide an insight into the Syrian paediatric population, who underwent rigid bronchoscopy during the current Syrian crisis (2011–2021). This study aims to describe the epidemiology, history, and complications presenting with FBA. Evaluating various aspects of FBA is an integral part of assessing efficacy in our management protocols.

## 2 Methods

### 2.1 Study design

This retrospective study was conducted at the Children's University Hospital and Al Mouwasat University hospital (AMUH), Damascus, Syria, between January (2011) and June (2018). AMUH is the only properly equipped hospital to perform paediatric rigid bronchoscopy, embracing most cases from Damascus.

### 2.2 Participants and procedures

**Figure 1:**
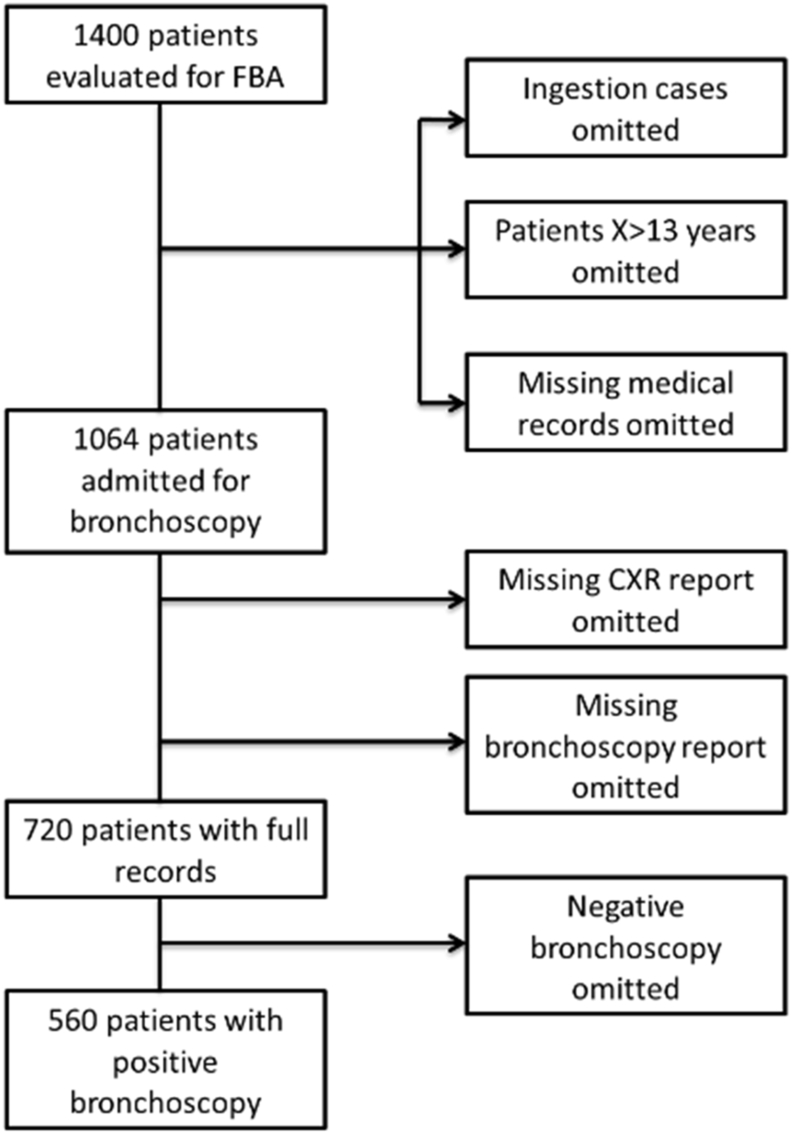
Flow diagram of patient population.

560 children with positive bronchoscopy of FBA were included in the study. The inclusion criteria for evaluation were complete medical records of children under the age of 13 years with positive bronchoscopy of FBA. Cases with bronchoscopy reports, but missing medical records were excluded, as either lost from the archives or mistaken by carers. Medical records with no bronchoscopy reports were excluded: contraindication for rigid bronchoscopy, 4 patients; withdrawn consent, 1 patient; discharged under carer responsibility, 9 patients. Carers who objected to bronchoscopy were told the risks and consequences and given fast track admission in case they had a change of mind. The authors believe the above 14 patients opted for private consultations and bronchoscopy as this was documented in the medical records. Negative bronchoscopy of FBA accounted for 160 cases, Figure 1 represents exclusion criteria.

### 2.3 Outcome measures

Variables included: age, gender, main complaint, detailed history, CXR report, and bronchoscopy report. The main complaint was divided into 3 categories: explicit history of FBA; suspicion of FBA with choking; and suspicion of FBA with recurrent respiratory tract infections. Explicit history is defined as a directly observed aspiration event by a witness or carer, while suspicion of FBA is an unwitnessed event. After clinical assessment and CXR, patients underwent rigid bronchoscopy under general anaesthesia then discharged, unless complicated, requiring hospitalization or the paediatric intensive care unit (PICU).

### 2.4 Ethical approval

Ethical approval was obtained from the Children's University Hospital and Al Mouwasat university hospital Institutional Review Board (IRB).

### 2.5 Statistical analysis

SPSS (Statistical package for social sciences) 25.0 program for Windows was used to calculate the data frequencies, percentages, means, and standard deviations (SD).

## 3 Results

### 3.1 Characteristics of participants

**Figure 2:**
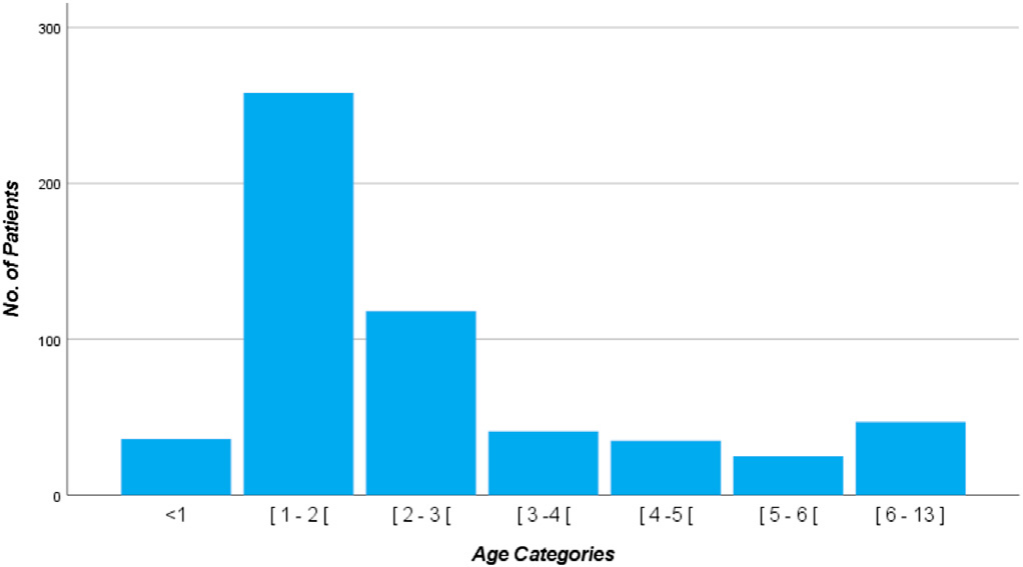
FBA by age incidence (years).

Of 560 patients included in the study, the median age was 1.8 years, the ages ranged from 1 month to 12 years, and the mean was 2.5 (±2.1) years. A male predominance 322 (57.5%) was found, with a male: female ratio of 1.4: 1. FBA peaked at the age of 1–2 years 258 (46.1%) where most incidences were between 1-3 years 376 (67.2%), and patients between 3-13 years 148 (26.5%) represented a minority (Figure 2).

### 3.2 History and examination

A total of 453 (80.9%) patients had an explicit history of FBA. Suspicion of FBA 107 (19.1%) was due to either coughing 78 (13.9%) or choking 29 (5.2%). On clinical examination the most prominent signs were dyspnea 320 (57.1%), wheezing 308 (55%), chest retractions 209 (37.35%), and decreased breath sounds 186 (33.9%). Normal auscultation was recorded in 107 (19.1%) patients (Table 1).

**Table 1:** Clinical Signs and symptoms.

		N	%
Main Complaint	Explicit history of FBA	453	80.9
	Suspicion of FBA + RTI + Coughing	78	13.9
	Suspicion of FBA + choking	29	5.2
Physical Examination	Dyspnea	320	57.1
	Chest retraction	209	37.3
	Stridor	62	11.1
	Fever	56	10.0
	Cyanosis	20	3.6
Chest Examination	Wheezing	308	55.0
	Normal sounds	107	19.1
	Decreased sounds	186	33.9
	Crepitations	111	19.8
	Diminished Sounds	3	0.5

### 3.3 Chest X-Ray

All 560 patients had a CXR; 115 (20.5%) were unremarkable. The majority of foreign bodies (FB) were radiolucent to x-ray beams, and 54 (9.6%) were radiopaque. Pulmonary hyperinflation 260 (46.4%), and pulmonary infiltrates 197 (35.2%) were the most frequent findings on CXR. Atelectasis 22 (3.9%) and tracheal deviation 25 (4.5%) were found in a minority of CXR (Table 2).

**Table 2:** Chest X-Ray findings.

	N	%
Normal	115	20.5
Hyperinflation	260	46.4
Infiltration	197	35.2
Radiopaque FB	54	9.6
Congestion	46	8.2
Tracheal Deviation	25	4.5
Atelectasis	22	3.9

### 3.4 Foreign body findings

Our study revealed that organic matter 467 (83.4%) was the most frequent FB. Seeds 273 (48.8%) and peanuts 101 (18%) were the most common form of organic matter. Other organic matter included: apple peel, carrot pieces, popcorn, and peas. Non-organic matter 71 (12.7%), metal and plastic objects, represented a minority of cases. 22 bronchoscopy reports did not record the type of FB as it may have been difficult to identify the matter.

Right main bronchus 255 (40.2%) was the most frequent site of lodgement, followed by left main bronchus 159 (28.4%), trachea 79 (14.1%), and larynx 47 (8.4%). 18 bronchoscopy reports did not record the site of FB (Table 3).

**Table 3:** Bronchoscopy findings.

		N	%
Type	Seed	273	48.8
	Peanut Other Organic matter Metallic body	101 93 42	18.0 16.6 7.5
	Plastic body	29	5.2
	Missing	22	3.9
Site	Supra vocal cord	17	3.0
	Sub vocal cord	30	5.4
	Trachea	79	14.1
	Right main bronchus Left main bronchus	225 159	40.2 28.4
	Missing	18	3.2

### 3.5 Complicated hospital stays

Respiratory tract infections (RTI), the commonest complication of FBA, mainly presented with lobar pneumonia. Post-bronchoscopy RTI included: lobar pneumonia 16 (2.9%), bronchiolitis 4 (0.7%), bronchopneumonia 3 (0.5%), pneumonia and pleurisy 1 (0.2%), bronchitis 1 (0.2%), and other infections 3 (0.5%). Pleural effusion after successful FB extraction occurred in 2 (0.4%) children. Complications of rigid bronchoscopy included: bronchial bleeding at the location of FB 1 (0.2%), post-operative stridor 1 (0.2%), and trauma to the palate 1 (0.2%). Anaesthesia attributed to a cardiac arrest event in a 1-year old child, who was managed with cardiopulmonary resuscitation. Rigid bronchoscopy was attempted and resulted in the successful removal of a seed FB located in the right main bronchus. The patient was then transferred to PICU and was managed there for 6 days, resulting in full recovery. 8 children had emergency bronchoscopies due to airway obstruction and deteriorating vital signs; 12 children required PICU. No mortality cases as a result of FB extraction were recorded (Table 4).

**Table 4:** Complicated hospital stays.

	N	%
Lobar pneumonia	16	2.9
Bronchiolitis	4	0.7
Bronchopneumonia	3	0.5
Pneumonia and pleurisy	1	0.2
Bronchitis	1	0.2
Other	3	0.5
Pleural effusion	2	0.4
Bronchial bleeding	1	0.2
Stridor	1	0.2
Palate trauma	1	0.2
Cardiorespiratory arrest	1	0.2
Mortality	0	0

## 4 Discussion

Significant morbidity and mortality can result from children inhaling objects obstructing the airways [1]. Diagnosing these accidents is crucial for the outcomes and the management of these patients. This study included patients, who underwent rigid bronchoscopy during a time of war and conflict. This provided an insight into the management of the biggest paediatric hospitals situated in the capital city of Syria, Damascus.

FBA is commonly seen between the ages 1–3 years representing over two-thirds of the population affected by FBA [15]. Our study revealed the peak incidence of FBA was among the age category 1–2 years. Our findings were in line with corresponding studies [8, 16, 17, 18, 19, 20]. The high prevalence of FBA at a young age could be explained by their lack of molar teeth; poor swallowing of food; their tendency to put objects in the mouth; using teething toys; crying, talking, or multitasking while eating; having immature protective laryngeal reflexes; having the desire to explore and interact with the surrounding world [21]. A slight male predominance was observed in our study 322 (57.5%) with a ratio of 1.4:1. Other studies showed that FBA was more common amongst boys than girls with a range of 54.8%–81.8% [8, 9, 16, 17, 22, 23, 24, 25, 26]. It's no secret that most boys are active, loud, rambunctious, have mischievous behaviour, and are adventurous in nature. Unfortunately labels boys as higher risk takers [17, 25, 26, 27, 28].

A definitive choking event was observed in most cases 453 (80.9%). On the other hand, studies revealed heterogeneous results ranging from 36.4% to 93.8% [8, 17]. This shows that an explicit history of an aspiration event may not always be present, delaying the diagnosis of FBA. Delay in diagnosis (over 30 days) was associated with more complications (95%) compared with early intervention [29]. Our patients presented with a wide range of clinical features, dyspnea 320 (57.1%), wheezing 308 (55%), chest retractions 209 (37.4%), and decreased breath sounds 186 (33.9%). A study comparing tracheal and bronchial FBA reported the diagnostic triad- coughing (96% and 98%), decreased breath sounds (28% and 63%), and wheezing (29% and 21%) as the most frequent findings [8].

CXR are frequently normal in FBA [24, 30, 31]. CXR reported in our study revealed, 115 (20.5%) normal, 260 (46.4%) hyperinflation, 197 (35.2%) infiltration, 25 (4.5%) tracheal deviation. The least common finding was atelectasis 22 (3.9%). On the other hand, a study on CXR radiological findings showed that 11.9% were normal, 38.8% showed atelectasis, 23.9% showed hyperinflation, and 14.9% showed deviation [22].

Flexible bronchoscopy offers a less invasive procedure compared with rigid bronchoscopy and is showing favourable outcomes in the removal of FB [32]. AMUH is only offering rigid bronchoscopy for the paediatric population and should consider the use of flexible bronchoscopy based on the benefits found in literature [12]. In our study, the right main bronchus 225 (40.2%) was the most common site of FB lodgement, which is comparable with other literature [30, 33, 34]. The right main bronchus has a wider lumen and less angulated branch compared with the left main bronchus, this anatomical structure facilitates easier lodgement of FB in the right main bronchus.

A literature review of 72 articles addressing food choking hazards stated the majority (94%) of FBA were of organic matter [35]. Our findings support the previous studies, where the majority of FB extracted were organic matter. Unaware of the consequences, infants and toddlers tend to put objects in their mouths and eat while going around their daily activities (play, run and laugh), which explains the high frequency of organic FBA among children [21]. Seeds represented almost half of FBA cases in this study. Arabs are the number one consumer of nuts and seeds, whether it's in their cooking or served as a pleasurable snack. Syrian women are known to enjoy eating seeds (called bizer) especially when meeting friends, watching T.V, and smoking hubble bubble. These women are also the mothers of our community and should be targeted in our awareness campaigns to educate them about the hazardous effects seeds can have upon their children when left within easy reach of their hands. These campaigns should aim to increase woman's awareness particularly when indulging in these snacks and educate women about disposing of hulls appropriately and quickly.

Despite the minority of FBA being of metallic origin, they still represent a concern. Recommendations for the prevention of FBA have been addressed through education and national policies. These policies include placing age restrictions on manufactured toys, by including minimum and maximum age use on the external packaging [35]. The government should implement these policies by reassessing choking hazards amongst manufacturers and reinforce mandatory labelling of products. This would significantly lower incidences of paediatric FBA in the community.

A comparison between the rate of mortality over 17 countries from Europe, Asia, Africa, and America was reported to be between 0 and 8.3% [36]. The number of deaths has declined over the past 50 years from 719 to 184 deaths between the dates 1968–2017 where laws, regulations, and guidelines were implemented in this period. Other factors contributing to this decline were unidentified [37]. No mortality incidences were recorded in our study, which could be attributed to early diagnosis and referral, quick intervention, and successful management. However, further analysis is pivotal to accepting these conclusions. Major complications include laryngeal oedema, failure to remove the FB, bronchospasm, hypoxic brain injury, pneumothorax, trauma to the airways, bronchial bleeding, and cardiac arrest [23, 33, 38, 39]. One of the complications faced by otorhinolaryngologists was intraoperative bleeding from the site of FB extraction. These cases did not require postponing and were dealt with during the procedures according to protocols. There was one case of mild trauma to the palate that was most likely due to the inexperience of the otorhinolaryngologist. Developing a platform for learning from mistakes, which may compromise patient safety, especially among junior doctors is crucial for avoiding future complications. Despite the low levels of complications, educating and increasing the awareness in the community amongst parents is crucial to avoid delays in intervention. Educational programs are being implemented in the community to target parents, carers, and children. The program aims to: teach children to avoid placing objects in their mouth, avoid giving children high-risk foods (seeds, sweets, fruits, and cashews), permitting food only at meal and snack times, and teaching basic life support from abdominal and chest thrusts to Heimlich manoeuvre. In a witnessed FBA or foreign body ingestion with acute complete airway obstruction, the Heimlich manoeuvre is the procedure of choice in children older than 1 year of age [40]. This manoeuvre is used when an individual is suffocating to relieve the obstruction and allows the flow of oxygen through the airways, preventing brain damage and death, as a result of prolonged oxygen depletion [2].

A major strength of this study lies in its large sample, recruited during a critical period, namely the devastating Syrian war. This paper is the first of its kind to report on the management of paediatric FBA in a country that has been severely burdened with economic collapse, mental health issues, and a poorly equipped health care system.

## 5 Limitations

The main limitation of the study is the retrospective aspect of data collection. Issues with data collection included: disorganised storage facilities, illegible handwriting, and subjective records. These problems could be addressed by handing out an admission questionnaire to carers on entry. In addition converting from medical records to electronic files, making the data more valid and accurate, and easier to access for future studies would eradicate most of the current weaknesses. A second limitation was that not all patients were considered in this study as some records were lost, and some patients admitted to the hospital withdrew and transferred to another hospital or private clinic for management. To overcome this problem a prospective multicentre study on a national level should be conducted to offer generalization. Another limitation was the unavailability of CT imaging. CT scans have a sensitivity of 100% and specificity of 98%, therefore preventing unnecessary bronchoscopies [5].

## 6 Conclusion

Paediatric FBA commonly presents amongst symptomatic males between 1-2 years, mostly seed aspirations that tends to lodge in the right main bronchus. The high number of seed aspiration by our children reflects a need for our society to realize the hazardous effects of our cultural seed eating habits. Syria must provide educational programs, particularly targeted at mothers, place warning labels on choking hazards, adopt advanced diagnostic techniques (CT), introduce flexible bronchoscopy to the management system, and increasing public awareness through mass media campaigns. Seeking to implement these recommendations will prevent further FBA and thus reduce the need for management and care of paediatric FBA in Syria.

## Declarations

### Author contribution statement

Fatema Mohsen: Conceived and designed the experiments; Analyzed and interpreted the data; Wrote the paper.

Batoul Bakkar: Performed the experiments; Wrote the paper.

Sara Melhem: Performed the experiments; Analyzed and interpreted the data.

Roula Altom: Performed the experiments.

Bisher Sawaf: Analyzed and interpreted the data.

Imad Alkhija: Contributed reagents, materials, analysis tools or data.

Louei Darjazini Nahas: Conceived and designed the experiments.

### Funding statement

This research did not receive any specific grant from funding agencies in the public, commercial, or not-for-profit sectors.

### Data availability statement

Data included in article/supplementary material/referenced in article.

### Declaration of interests statement

The authors declare no conflict of interest.

### Additional information

No additional information is available for this paper.
